# Supplementary material for: Genome-Inspired Chemical Exploration of Marine Fungus Aspergillus fumigatus MF071
Source: Mar Drugs. 2020 Jul 6;18(7):352. doi: 10.3390/md18070352 (PMC7401266; doi:10.3390/md18070352)
Supplement: Supplementary file 1 [file marinedrugs-18-00352-s001.pdf]

Supplementary materials

Genome-inspired chemical exploring of marine fungus *Aspergillus fumigatus* MF071

Jianying Han [1,3], Miaomiao Liu [1], Ian D. Jenkins [1], Xueting Liu [2], Lixin Zhang [2,3], Ronald J. Quinn [1,*] and Yunjiang Feng [1,*]

[1] Griffith Institute for Drug Discovery, Griffith University, Brisbane, QLD 4111, Australia; jianying.han@griffithuni.edu.au (J.H.); miaomiao.liu@griffith.edu.au (M.L.); i.jenkins@griffith.edu.au (I.D.J.)

[2] State Key Laboratory of Bioreactor Engineering, East China University of Science and Technology, Shanghai 200237, China; liuxueting@ecust.edu.cn (X.L.); lxzhang@ecust.edu.cn (L.Z.)

[3] Key Laboratory of Pathogenic Microbiology and Immunology, Institute of Microbiology, Chinese Academy of Sciences, Beijing 100101, China;

* Correspondence: r.quinn@griffith.edu.au (R.J.Q.), y.feng@griffith.edu.au (Y.F.); Tel.: +61-7-3735-6006 (R.J.Q.), +61-7-3735-8367 (Y.F.)

Table of Contents

Figure S1 HRESIMS spectrum of **1**
Figure S2 ^{1}H NMR (800 MHz, DMSO-d_6) spectra of **1**
Figure S3 HSQC (800 MHz, DMSO-d_6) spectrum of **1**
Figure S4 HMBC (800 MHz, DMSO-d_6) spectrum of **1**
Figure S5 HRESIMS spectrum of **2**
Figure S6 ^{1}H NMR (800 MHz, DMSO-d_6) spectra of **2**
Figure S7 ^{1}H-^{1}H COSY (800 MHz, DMSO-d_6) spectrum of **2**
Figure S8 HSQC (800 MHz, DMSO-d_6) spectrum of **2**
Figure S9 HMBC (800 MHz, DMSO-d_6) spectrum of **2**
Figure S10 HRESIMS spectrum of **4**
Figure S11 ^{1}H NMR (800 MHz, DMSO-d_6) spectra of **4**
Figure S12 ^{13}C NMR (200 MHz, DMSO-d_6) spectrum of **4**
Figure S13 HSQC (800 MHz, DMSO-d_6) spectrum of **4**
Figure S14 HMBC (800 MHz, DMSO-d_6) spectrum of **4**
Figure S15 HRESIMS spectrum of **10**
Figure S16 ^{1}H NMR (800 MHz, DMSO-d_6) spectra of **10**
Figure S17 ^{13}C NMR (200 MHz, DMSO-d_6) spectrum of **10**
Figure S18 HSQC (800 MHz, DMSO-d_6) spectrum of **10**
Figure S19 HMBC (800 MHz, DMSO-d_6) spectrum of **10**
Figure S20 Organization of the fusidane-type antibiotic helvolic acid BGC (*hel*) (**A**) and proposed biosynthetic pathways for helvolic acid and helvolinic acid (**B**).
Figure S21 Representatives of prenylated indole alkaloids
Table S1 The calculated ^{13}C NMR data for two possible isomers (19*S*) and (19*R*) of compound **1** and DP4 analysis
Table S2 Deduced functions of ORFs in fumitremorgins BGC (*ftm*) from MF071
Table S3 Deduced functions of ORFs in pseurotins BGC (*pso*) from MF071
Table S4 Deduced functions of ORFs in fumigaclavines BGC (*fga*) from MF071
Table S5 Deduced functions of ORFs in helvolic acid BGC (*hel*) from MF071

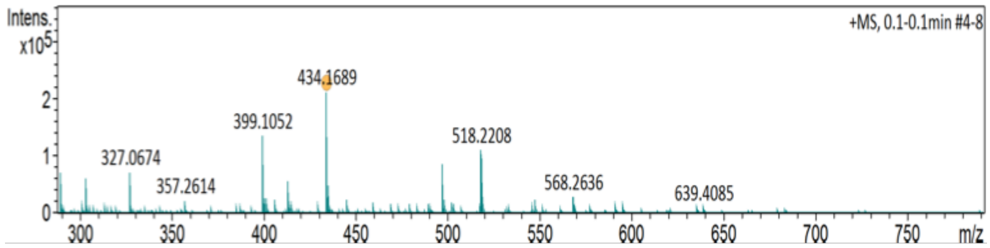

Figure S1 HRESIMS spectrum of **1**

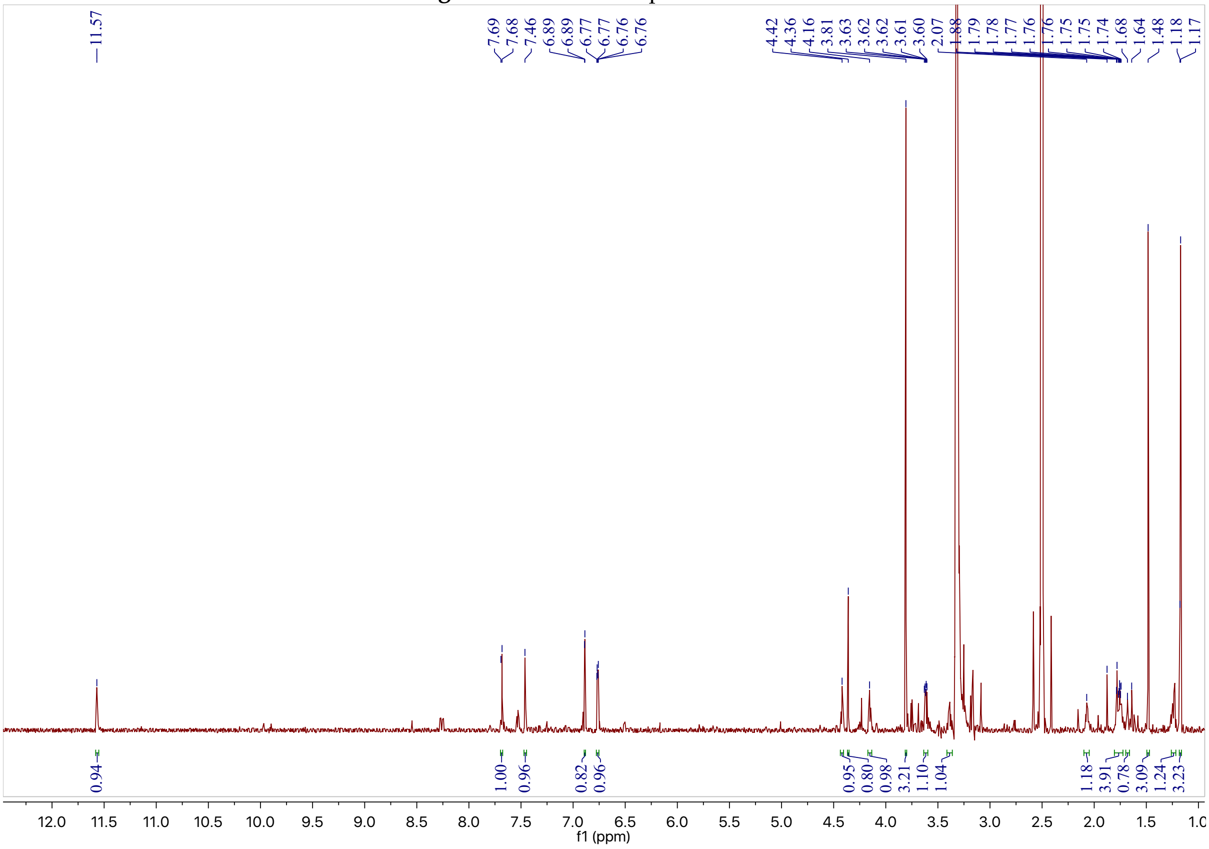

Figure S2 ^{1}H NMR (800 MHz, DMSO-d_6) spectra of **1**

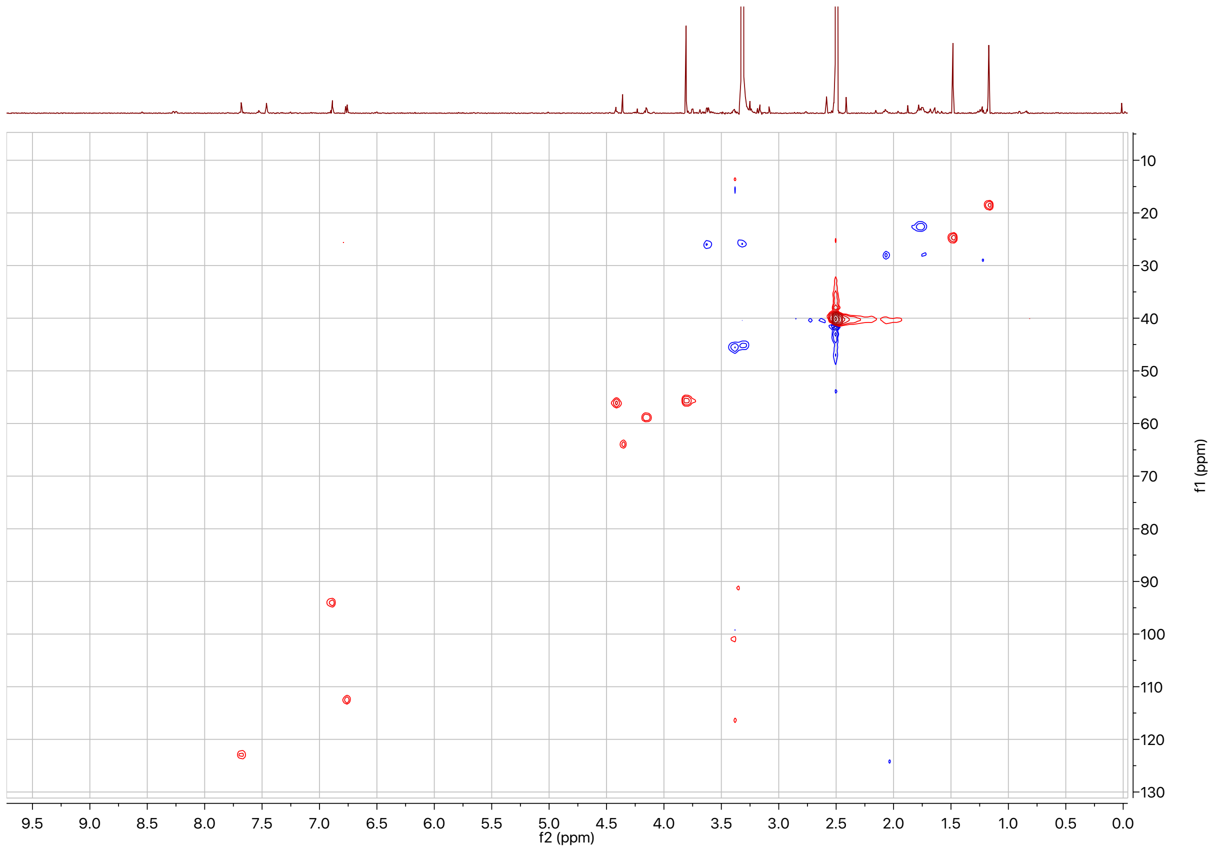

Figure S3 HSQC (800 MHz, DMSO-d_6) spectrum of **1**

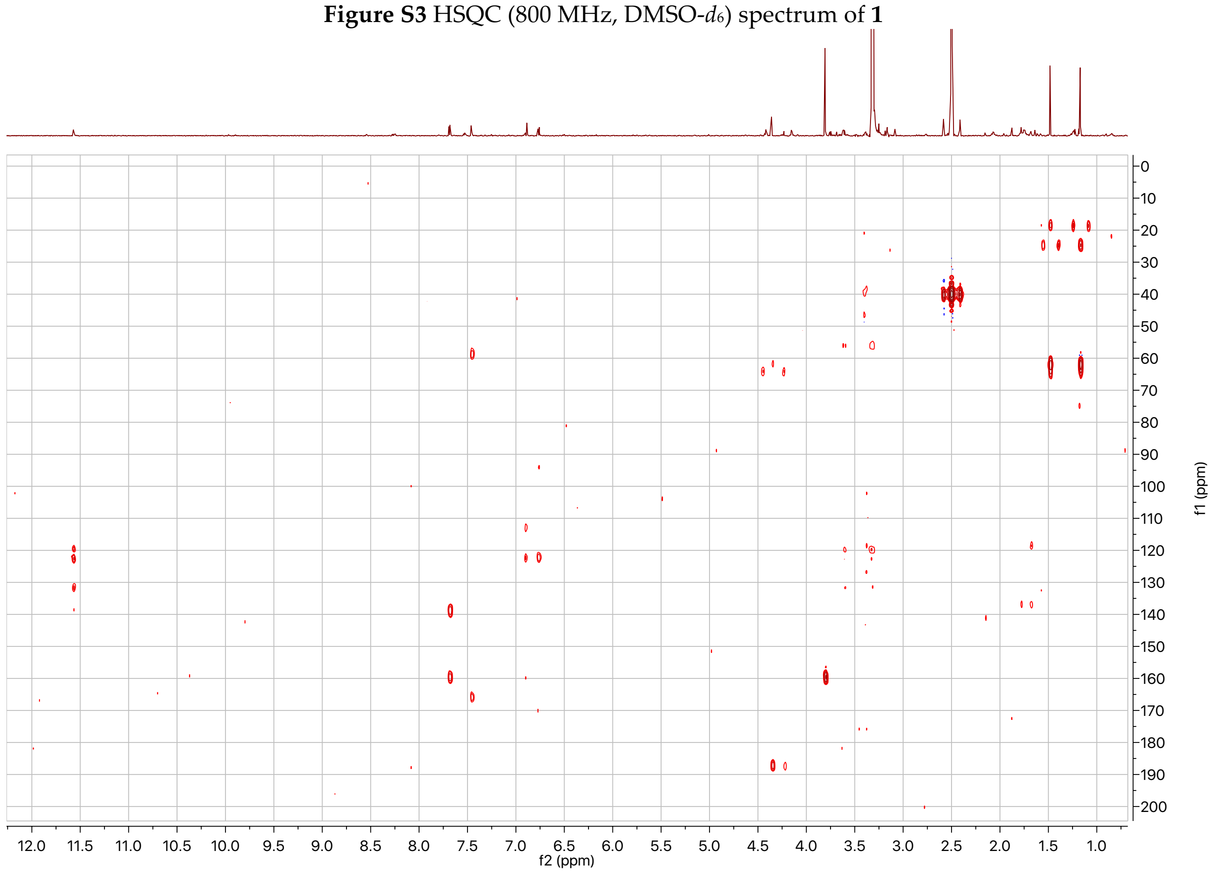

Figure S4 HMBC (800 MHz, DMSO-d_6) spectrum of **1**

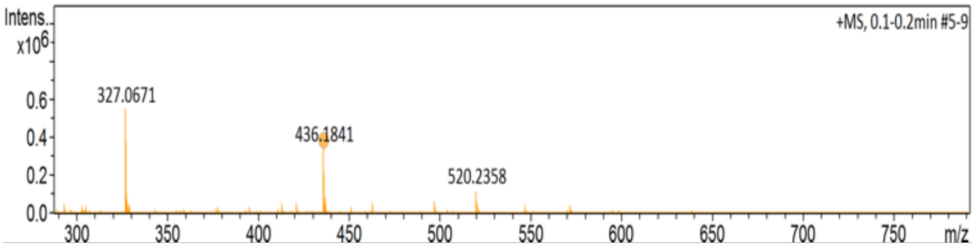

Figure S5 HRESIMS spectrum of **2**

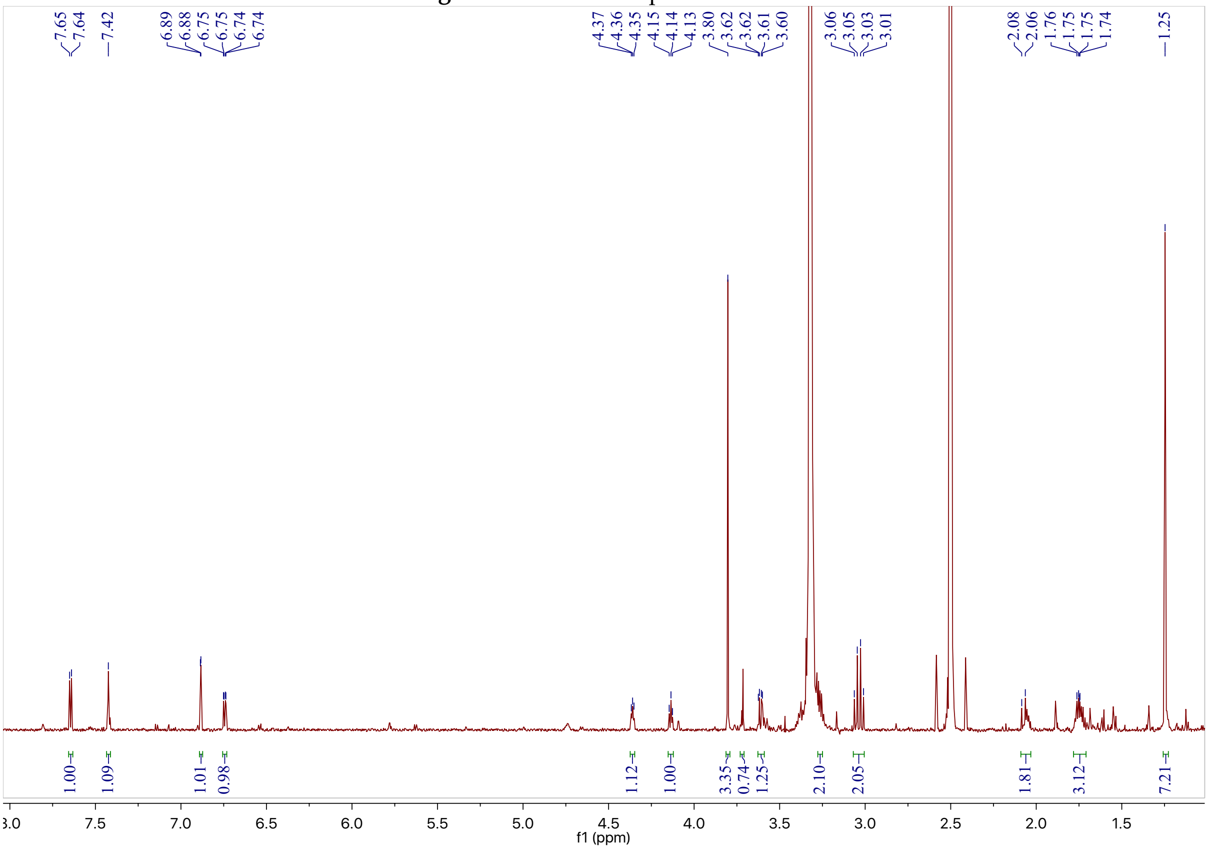

Figure S6 ^{1}H NMR (800 MHz, DMSO-d_6) spectra of **2**

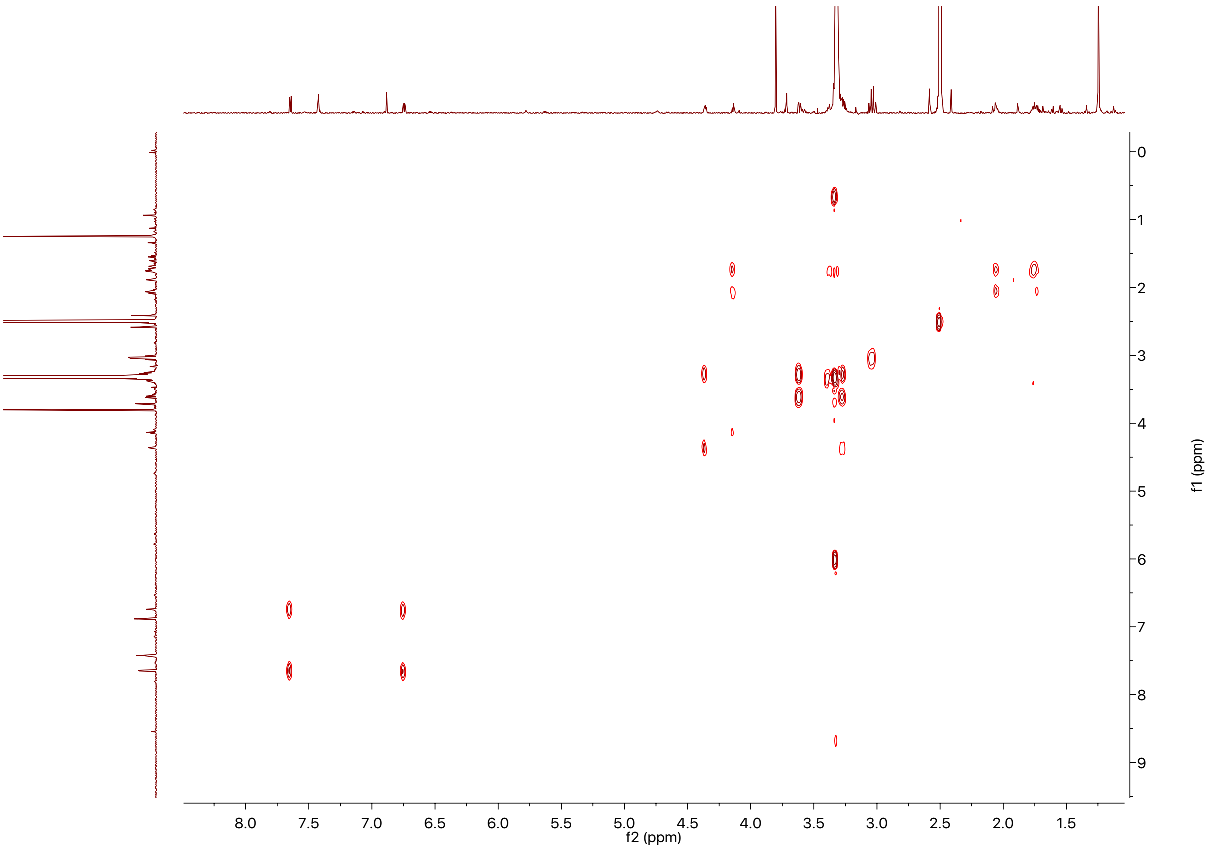

Figure S7 ^{1}H-^{1}H COSY (800 MHz, DMSO-d_6) spectrum of **2**

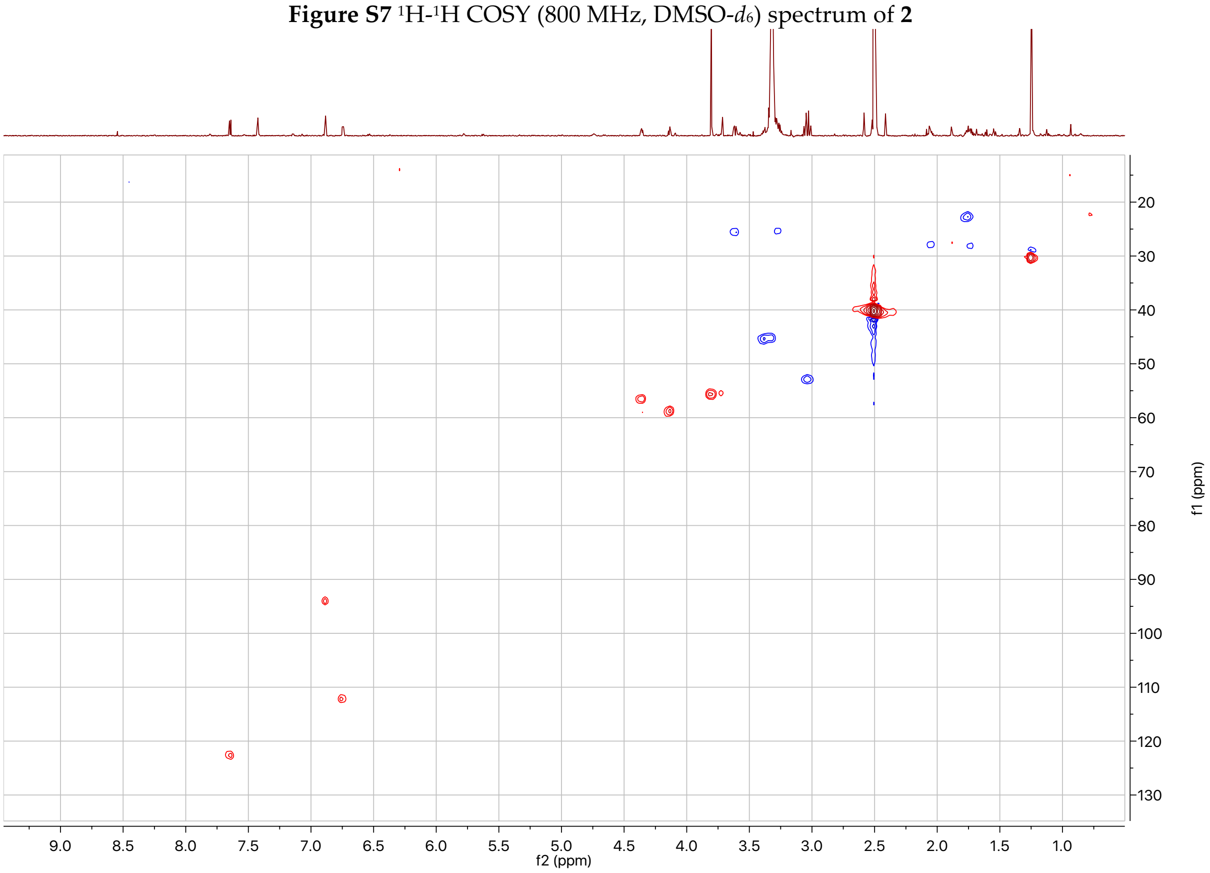

Figure S8 HSQC (800 MHz, DMSO-d_6) spectrum of **2**

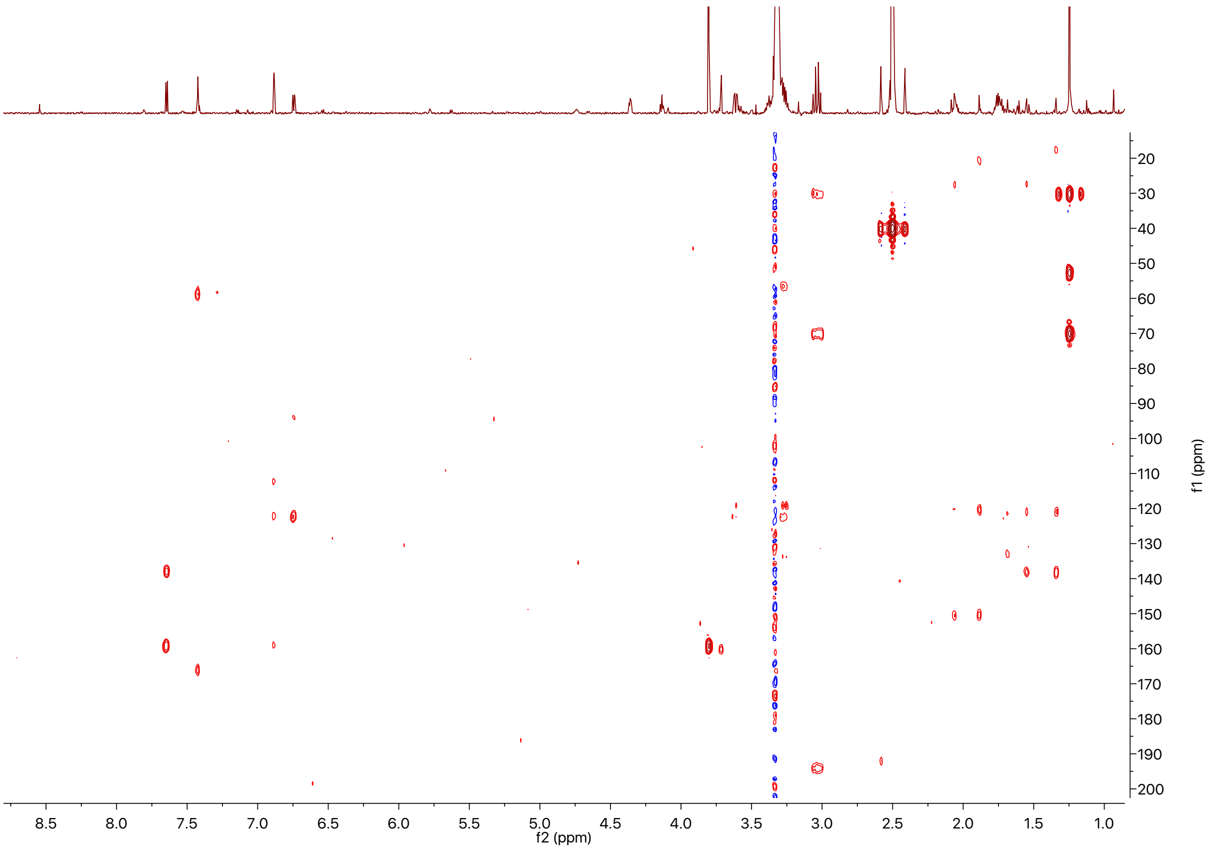

Figure S9 HMBC (800 MHz, DMSO-d_6) spectrum of **2**

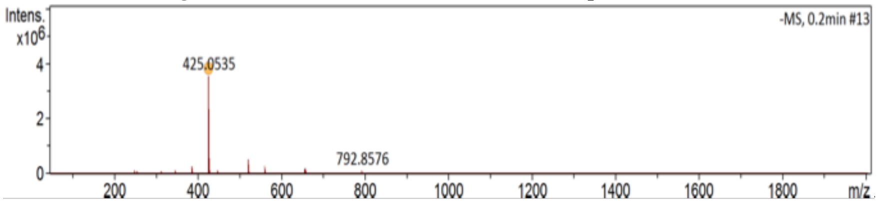

Figure S10 HRESIMS spectrum of **4**

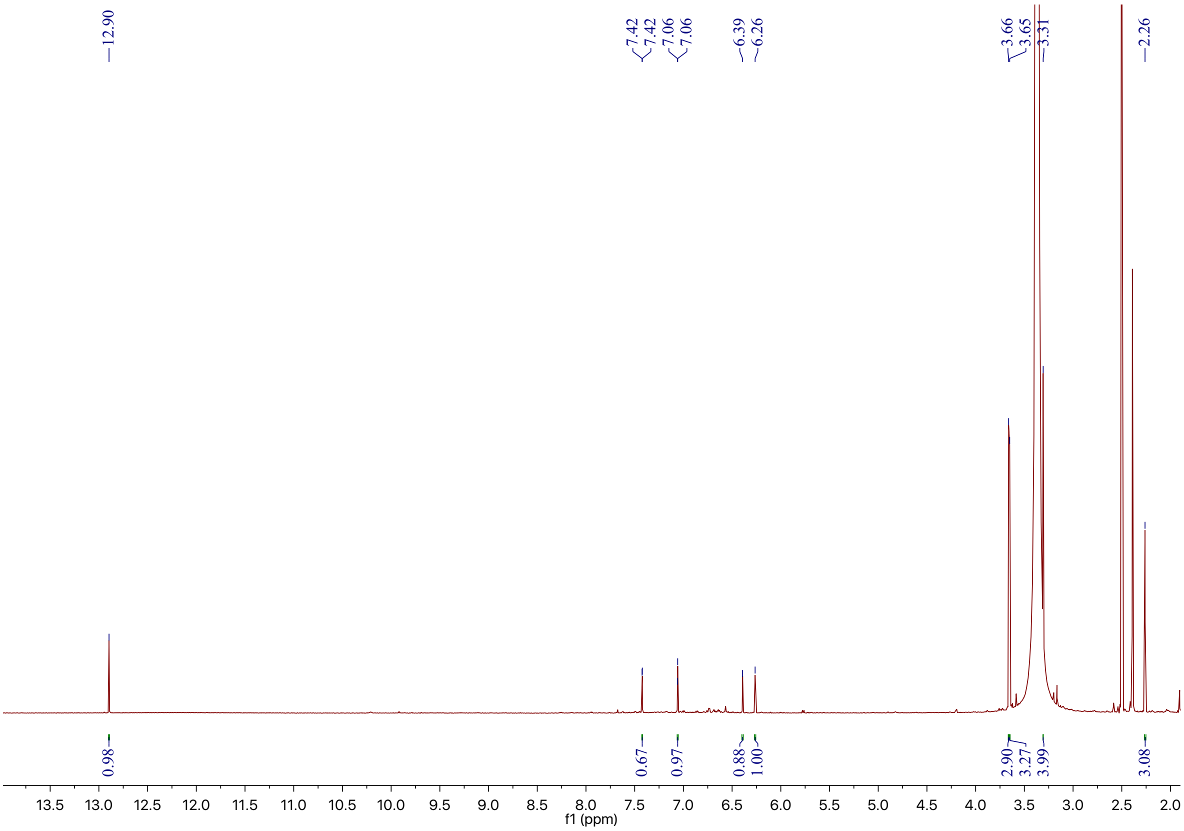

Figure S11 ^{1}H NMR (800 MHz, DMSO-d_6) spectra of **4**

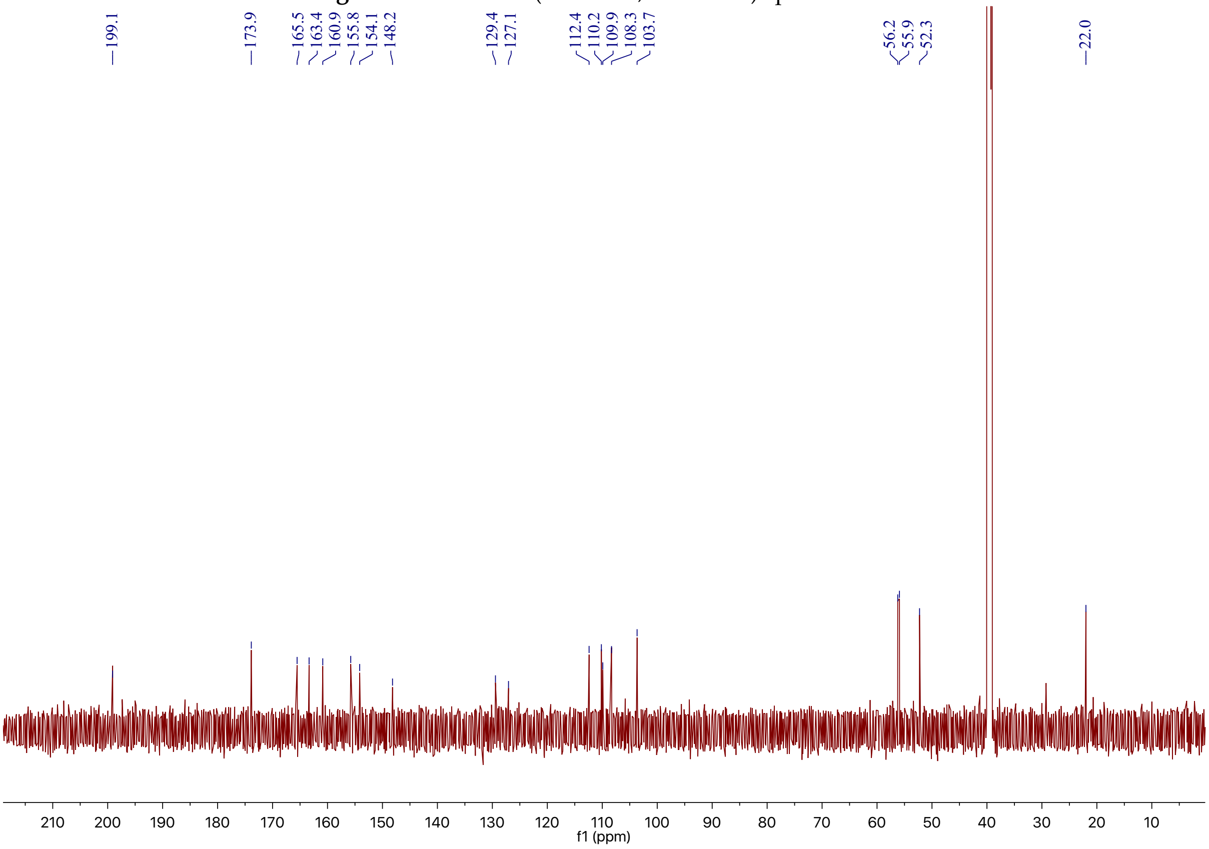

Figure S12 ^{13}C NMR (200 MHz, DMSO-d_6) spectrum of **4**

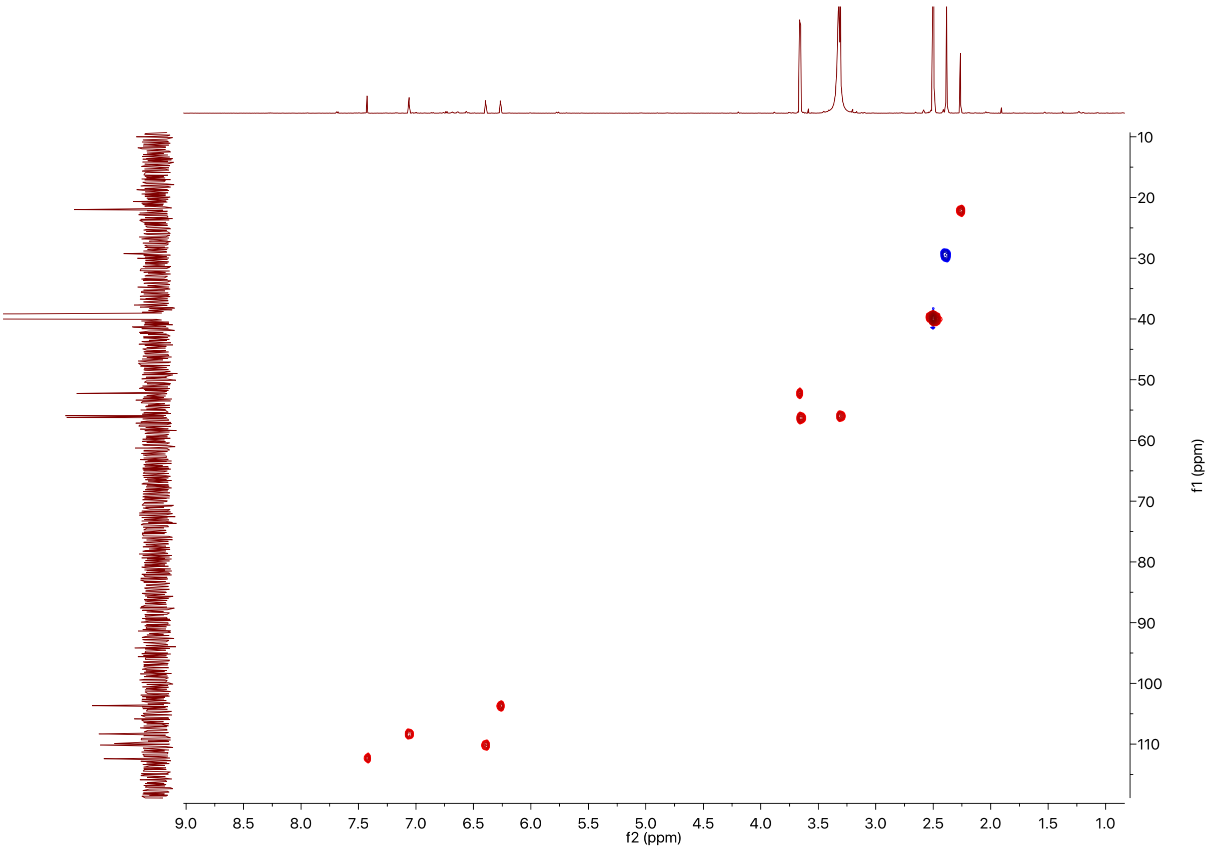

Figure S13 HSQC (800 MHz, DMSO-d_6) spectrum of **4**

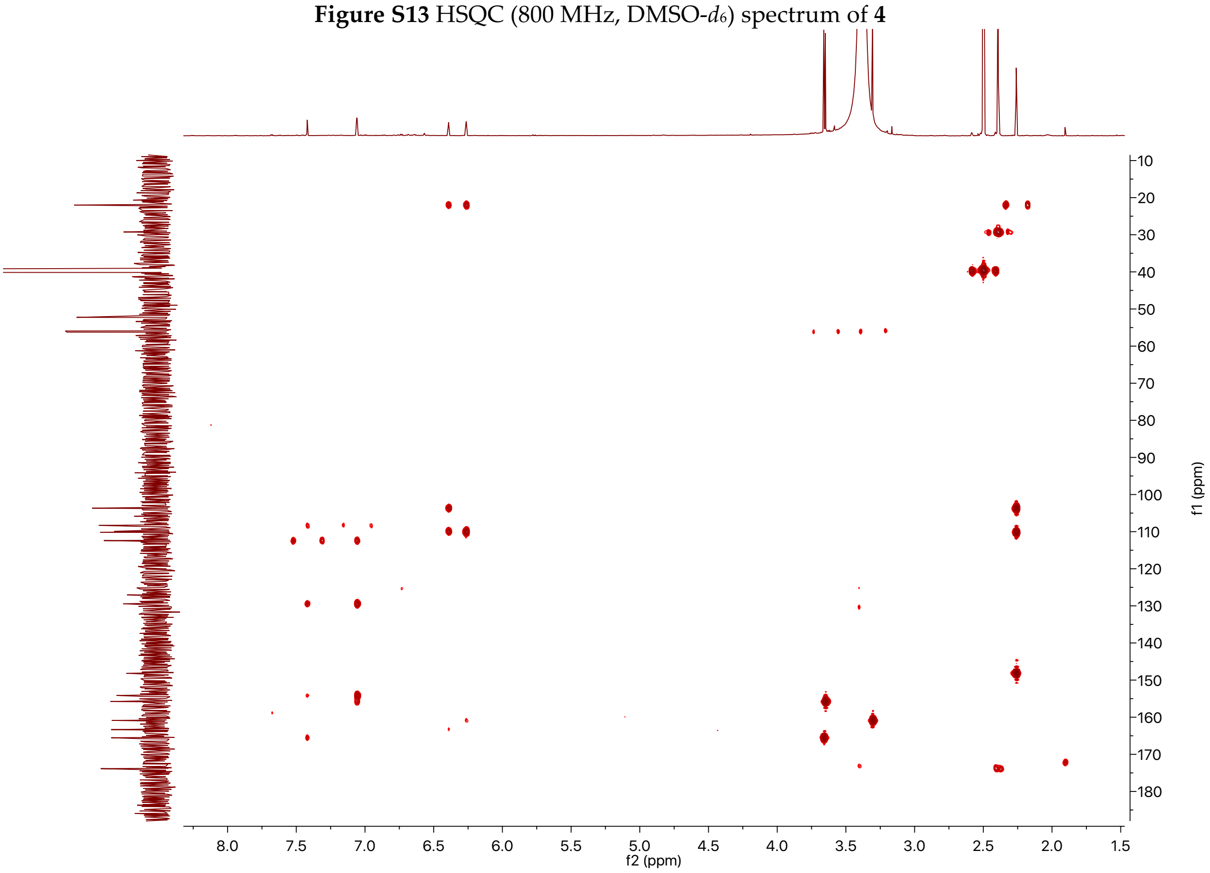

Figure S14 HMBC (800 MHz, DMSO-d_6) spectrum of **4**

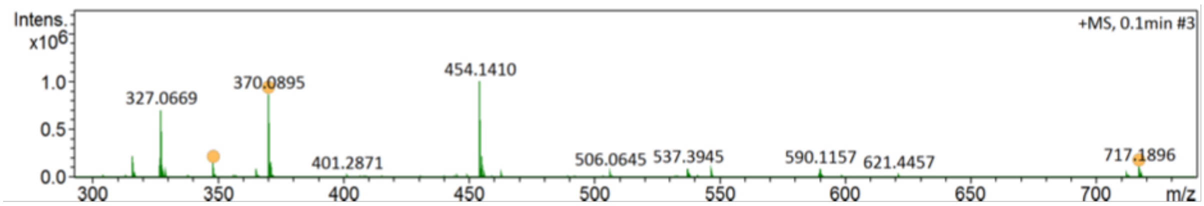

Figure S15 HRESIMS spectrum of compound **10**

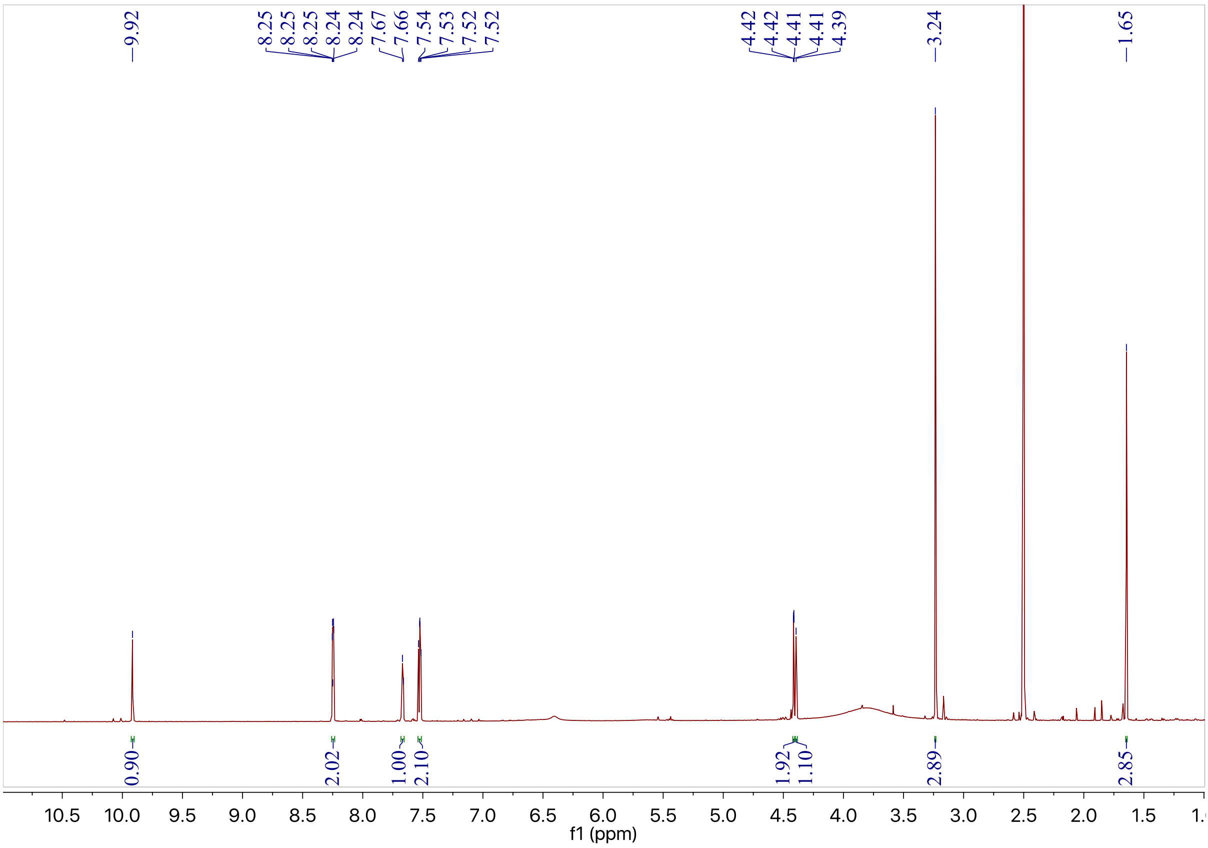

Figure S16 ^{1}H NMR (800 MHz, DMSO-d_6) spectra of **10**

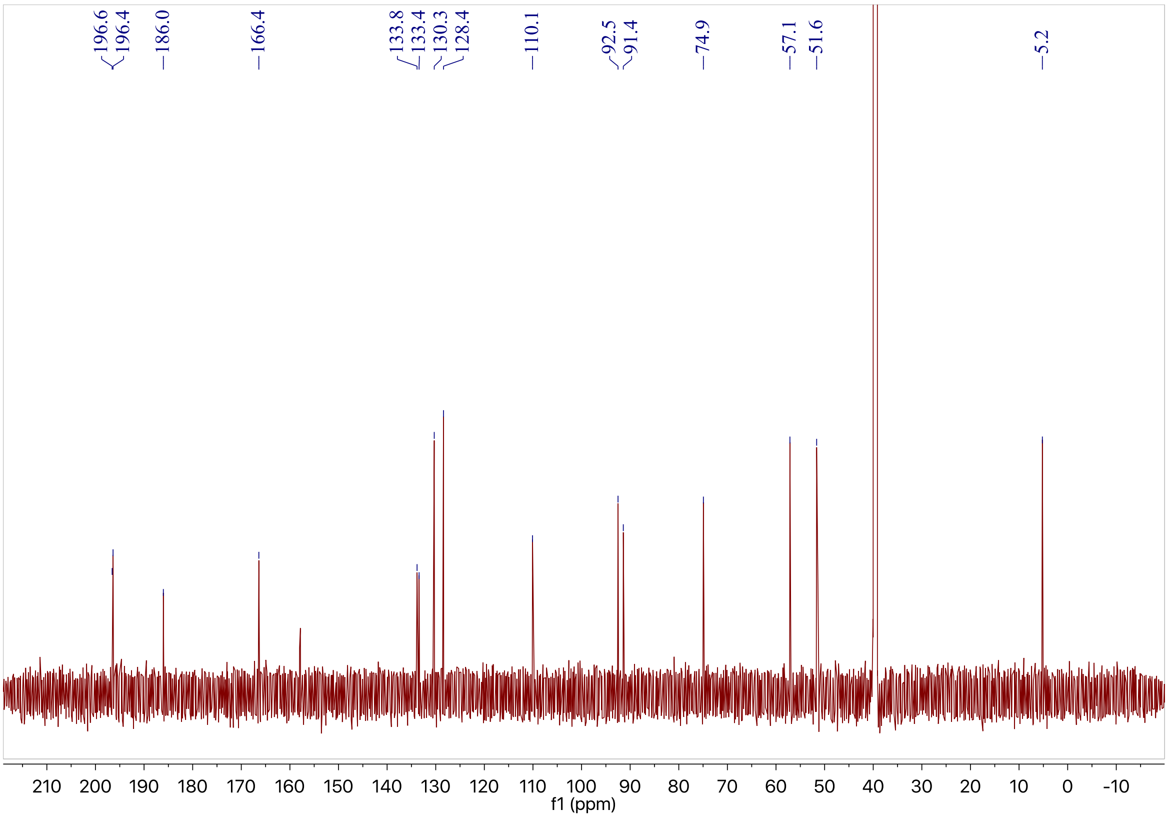

Figure S17 ^{13}C NMR (200 MHz, DMSO-d_6) spectrum of **10**

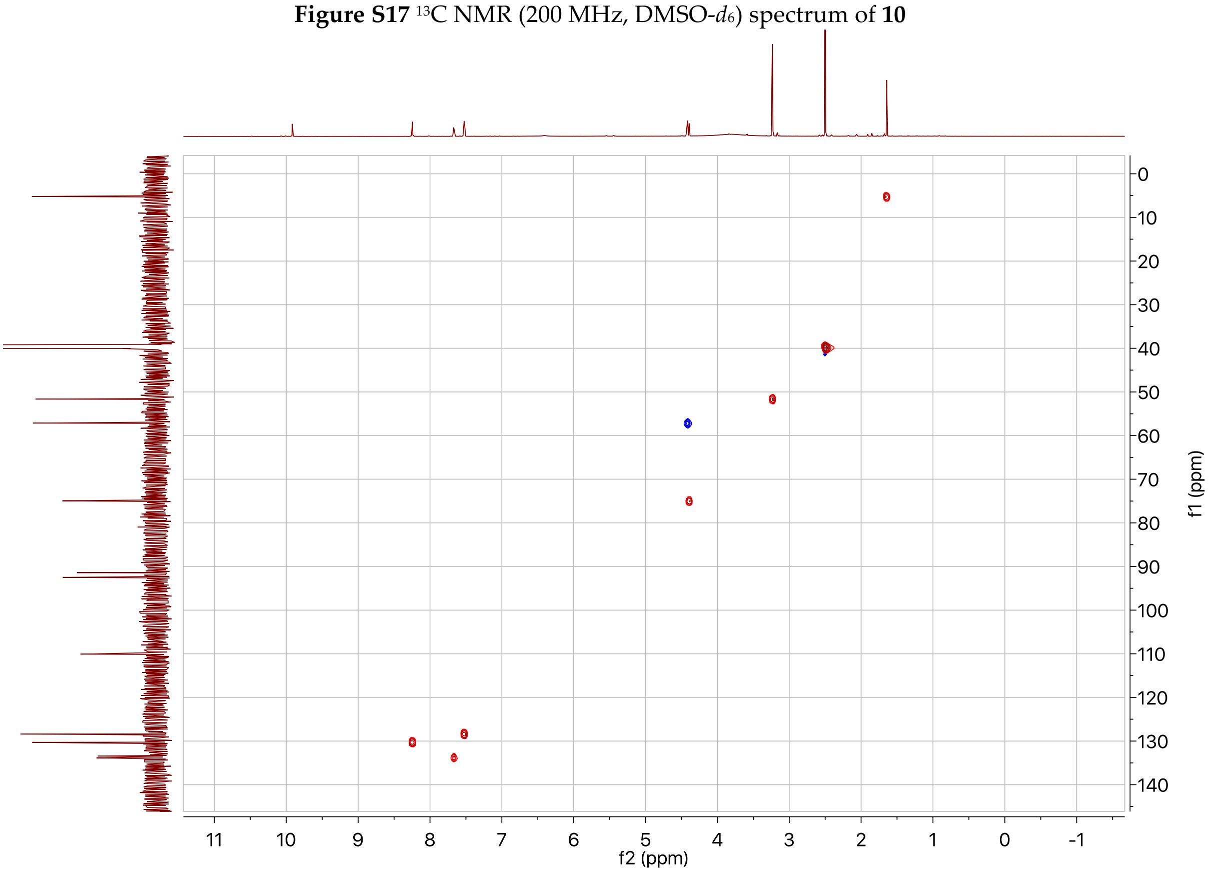

Figure S18 HSQC (800 MHz, DMSO-d_6) spectrum of **10**

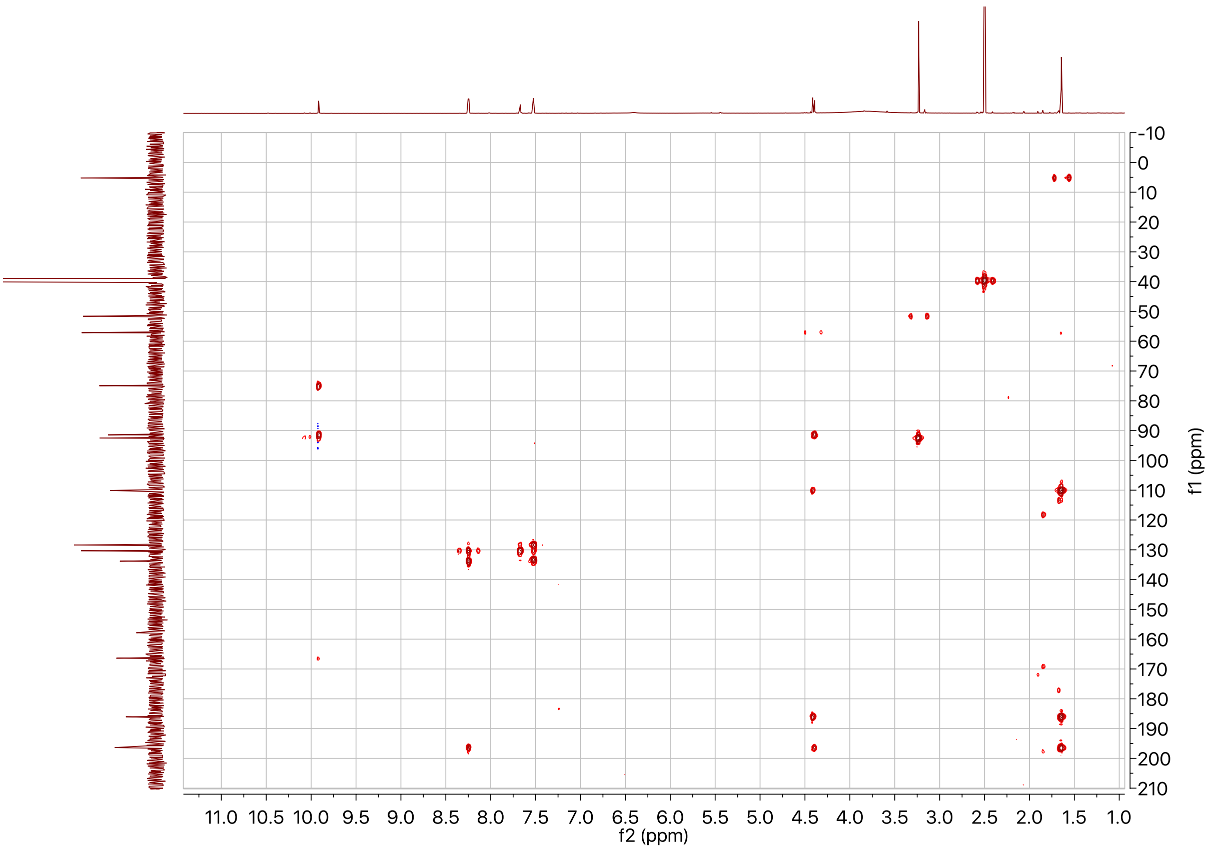

Figure S19 HMBC (800 MHz, DMSO-*d*6) spectrum of **10**

A

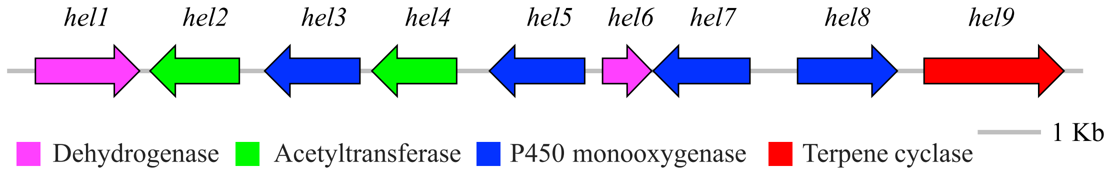

B

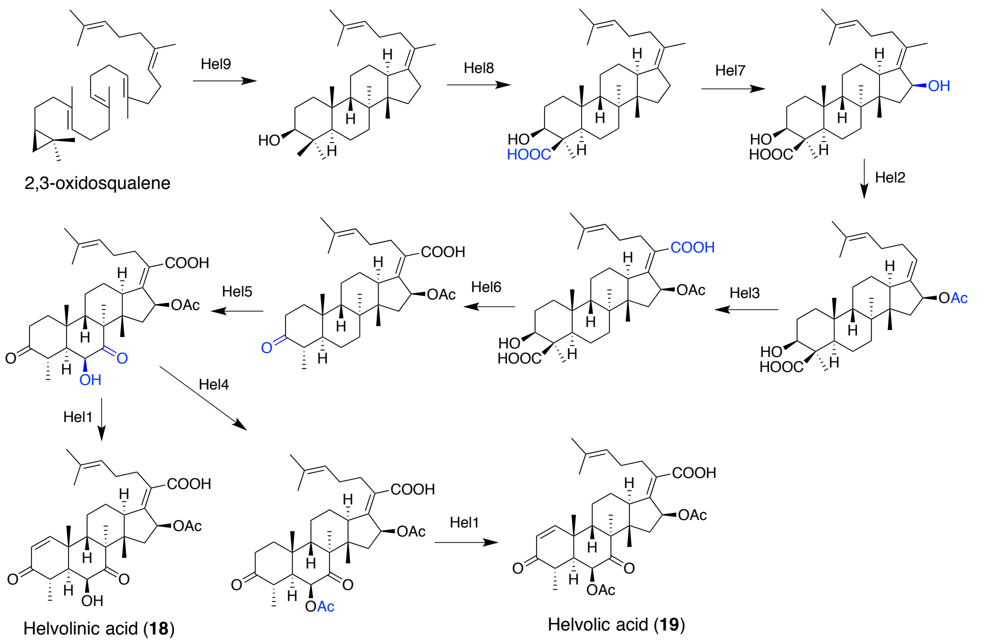

Figure S20 Organization of the fusidane-type antibiotic helvolic acid BGC (*hel*) (**A**) and proposed biosynthetic pathways for helvolic acid and helvolinic acid.

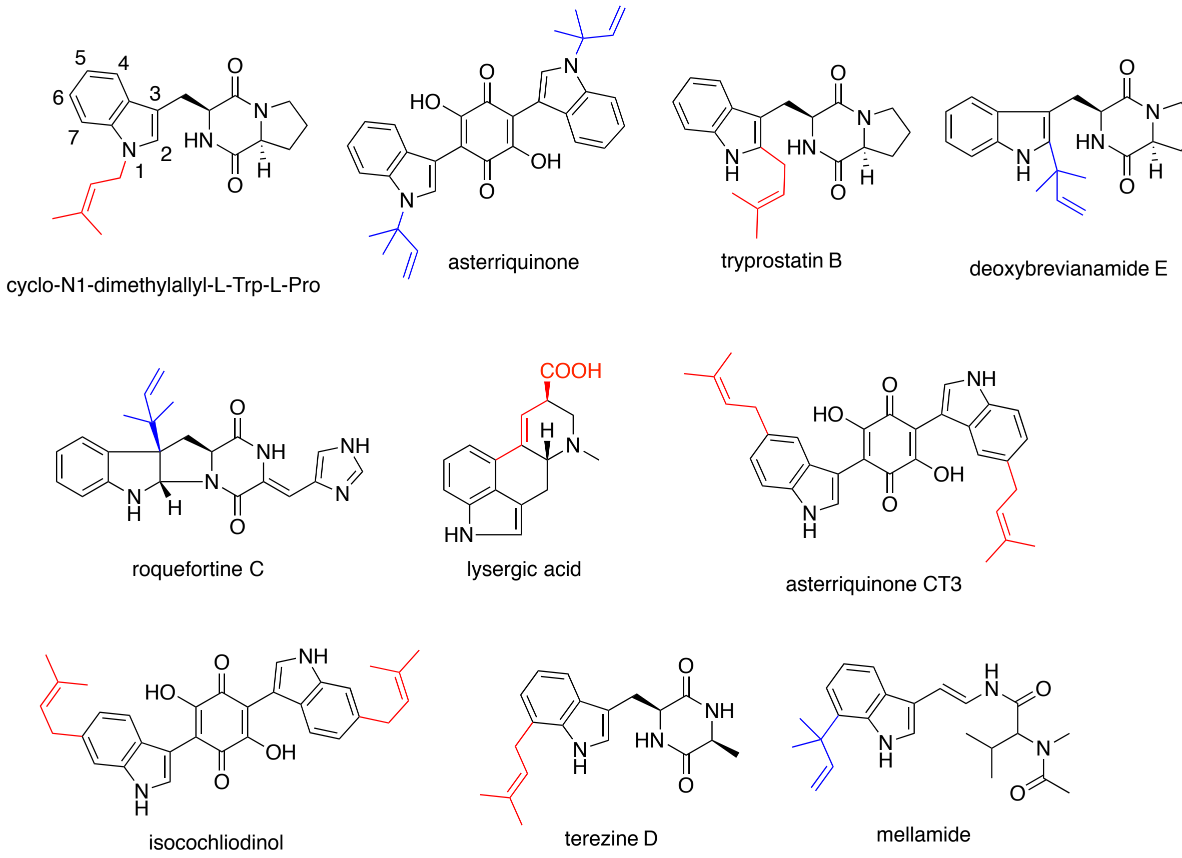

Figure S21 Representatives of prenylated indole alkaloids.

Table S1 The calculated ^{13}C NMR data for two possible isomers (19*S*) and (19*R*) of compound **1** and DP4 analysis

No.	δexp	δcal		δscal		Error		t distribution		Probability	
		**(19*S*)**	**(19*R*)**	**(19*S*)**	**(19*R*)**	**(19*S*)**	**(19*R*)**	**(19*S*)**	**(19*R*)**	**(19*S*)**	**(19*R*)**
2	131.7	138.1	137.8	131.5	134.8	-0.2	3.1	0.527	0.899	0.473	0.101
3	119.8	133.5	130.5	127.0	127.5	7.2	7.7	0.995	0.997	0.005	0.003
3a	122.4	129.5	128.0	123.1	125.0	0.7	2.6	0.611	0.856	0.389	0.144
4	123.0	133.5	126.9	127.0	123.9	4.0	0.9	0.945	0.644	0.055	0.356
5	112.5	119.4	119.2	113.1	116.2	0.6	3.7	0.602	0.930	0.398	0.070
6	159.8	169.5	168.8	162.4	165.9	2.6	6.1	0.854	0.988	0.146	0.012
7	94.1	90.9	93.5	85.1	90.4	-9.0	-3.7	0.999	0.932	0.001	0.068
7a	138.7	144.2	143.2	137.5	140.3	-1.2	1.6	0.694	0.748	0.306	0.252
8	26.0	29.4	33.0	24.6	29.8	-1.4	3.8	0.725	0.935	0.275	0.065
9	56.1	65.3	65.8	59.9	62.6	3.8	6.5	0.937	0.992	0.063	0.008
11	167.2	171.7	175.1	164.6	172.2	-2.6	5.0	0.859	0.974	0.141	0.026
12	58.9	64.8	61.4	59.5	58.2	0.6	-0.7	0.594	0.614	0.406	0.386
13	28.1	33.7	30.4	28.8	27.1	0.7	-1.0	0.623	0.663	0.377	0.337
14	22.6	25.9	26.4	21.1	23.1	-1.5	0.5	0.733	0.579	0.267	0.421
15	45.5	49.1	48.9	44.0	45.7	-1.5	0.2	0.742	0.526	0.258	0.474
17	165.9	170.9	170.1	163.7	167.2	-2.2	1.3	0.815	0.709	0.185	0.291
18	187.2	193.4	193.1	185.9	190.3	-1.3	3.1	0.713	0.895	0.287	0.105
19	63.9	68.2	73.6	62.7	70.5	-1.2	6.6	0.686	0.992	0.314	0.008
20	61.8	69.3	69.2	63.8	66.0	2.0	4.2	0.797	0.954	0.203	0.046

21	24.7	26.1	26.5	21.4	23.2	-3.3	-1.5	0.913	0.734	0.087	0.266
22	18.6	18.8	20.5	14.2	17.2	-4.4	-1.4	0.959	0.726	0.041	0.274
$O\underline{C}H_3$	55.6	**55.6**	55.6	50.3	52.4	-5.3	-3.2	0.979	0.907	0.021	0.093
Product of Probability										7.46E-21	6.47E-24
Bayes's theorem probability (%)										99.9	0.1

Table S2 Deduced functions of ORFs in fumitremorgins BGC (*ftm*) from MF071

Protein	Homology	Proposed function	Origin	Accession No.	Identity (%)
Ftm1	FtmA	nonribosomal peptide synthetase	*A. fumigatus* BM939	B9WZX0	89
Ftm2	FtmC	cytochrome P450	*A. fumigatus* BM939	XP_747185	100
Ftm3	FtmD	O-methyltransferase	*A. fumigatus* NRRL 181	XP_001261648	93
Ftm4	FtmB	prenyltransferase	*A. fumigatus* BM939	B9WZX3	82
Ftm5	FtmE	cytochrome P450	*A. fumigatus* Af293	XP_747182	99
Ftm6	FtmF	oxygenase	*A. fumigatus* BM939	B9WZX5	100
Ftm7	FtmG	cytochrome P450	*A. fumigatus* BM939	B9WZX6	95
Ftm8	FtmH	prenyltransferase	*A. fumigatus* BM939	B9WZX7	99

Table S3 Deduced functions of ORFs in pseurotins BGC (*pso*) from MF071

Protein	Homology	Proposed function	Origin	Accession No.	Identity (%)
Pso1	PsoF	Dual-functional monooxygenase/methyltransferase	A. fumigatus Af293	XP_747160	96
Pso2	PsoG	methionine aminopeptidase	A. fumigatus Af293	XP_747159	100
Pso3	PsoB	alpha/beta hydrolase	A. fumigatus Af293	XP_747152	100
Pso4	PsoA	hybrid PKS-NRPS enzyme	A. fumigatus Af293	XP_747151	97
Pso5	PsoC	methyltransferase	A. fumigatus Af293	XP_747150	100
Pso6	PsoD	cytochrome P450	A. fumigatus Af293	XP_747149	89
Pso7	PsoE	glutathione S-transferase	A. fumigatus Af293	XP_747147	100

Table S4 Deduced functions of ORFs in fumigaclavines BGC (*fga*) from MF071

Protein	Homology	Proposed function	Origin	Accession No.	Identity (%)
Fga1	FgaMT	4-dimethylallyltryptophan N-methyltransferase	*A. fumigatus* Af293	XP_756143	100
Fga2	FgaOx1	FAD binding oxidoreductase	*A. fumigatus* Af293	XP_756142	100
Fga3	FgaPT2	L-tryptophan dimethylallyl transferase	*A. fumigatus* Af293	XP_756141	88
Fga4	FgaCat	catalase Cat	*A. fumigatus* Af293	XP_756140	100
Fga5	FgaAT	O-acetyltransferase	*A. fumigatus* Af293	XP_756139	99
Fga6	FgaP450-2	cytochrome P450	*A. fumigatus* Af293	XP_756138	100

Fga7	FgaDH	dehydrogenase/oxidoreductase	*A. fumigatus* Af293	XP_756137	100
Fga8	FgaPT1	dimethylallyl tryptophan synthase	*A. fumigatus* Af293	XP_756136	100
Fga9	FgaP450-1	cytochrome P450	*A. fumigatus* Af293	XP_756135	100
Fga10	FgaFs	festuclavine dehydrogenase easG	*A. fumigatus* Af293	XP_756134	100
Fga11	FgaOx3	chanoclavine-i aldehyde reductase	*A. fumigatus* Af293	4QNW_A	100

Table S5 Deduced functions of ORFs in helvolic acid BGC (*hel*) from MF071

Protein	Homology	Proposed function	Origin	Accession No.	Identity (%)
Hel1	HelE	dehydrogenase	*A. fumigatus* Af293	XP_751348	94
Hel2	HelD2	acetyltransferase	*A. fumigatus* Af293	XP_751349	93
Hel3	HelB4	cytochrome P450	*A. fumigatus* Af293	XP_751350	95
Hel4	HelD1	acetyltransferase	*A. fumigatus* Af293	XP_751351	89
Hel5	HelB3	cytochrome P450	*A. fumigatus* Af293	XP_751352	91
Hel6	HelC	dehydrogenase	*A. fumigatus* Af293	XP_751353	87
Hel7	HelB2	cytochrome P450	*A. fumigatus* Af293	XP_751354	99
Hel8	HelB1	cytochrome P450	*A. fumigatus* Af293	XP_751355	100
Hel9	HelA	squalene-hopene cyclase	*A. fumigatus* Af293	XP_751356	100
